# Properties of graphene deposited on GaN nanowires: influence of nanowire roughness, self-induced nanogating and defects

**DOI:** 10.3762/bjnano.12.47

**Published:** 2021-06-22

**Authors:** Jakub Kierdaszuk, Piotr Kaźmierczak, Justyna Grzonka, Aleksandra Krajewska, Aleksandra Przewłoka, Wawrzyniec Kaszub, Zbigniew R Zytkiewicz, Marta Sobanska, Maria Kamińska, Andrzej Wysmołek, Aneta Drabińska

**Affiliations:** 1 Faculty of Physics, University of Warsaw, Warsaw, Poland; 2 University of Cadiz, Department of Material Science and Metallurgy Engineering and Inorganic Chemistry, Cadiz, Spain; 3 Łukasiewicz - Institute of Microelectronics and Photonics, Warsaw, Poland; 4 CENTERA Laboratories, Institute of High Pressure Physics PAS, Warsaw, Poland; 5 Institute of Optoelectronics, Military University of Technology, Warsaw, Poland; 6 Institute of Physics, Polish Academy of Sciences, Al. Lotnikow 32/46, 02-668, Warsaw, Poland

**Keywords:** carrier concentration, gallium nitride, graphene, nanowires, Raman spectroscopy, scattering on defects, strain

## Abstract

We present detailed Raman studies of graphene deposited on gallium nitride nanowires with different variations in height. Our results indicate that different density and height of nanowires impact graphene properties such as roughness, strain, and carrier concentration as well as density and type of induced defects. Tracing the manifestation of those interactions is important for the application of novel heterostructures. A detailed analysis of Raman spectra of graphene deposited on different nanowire substrates shows that bigger differences in nanowires height increase graphene strain, while a higher number of nanowires in contact with graphene locally reduces the strain. Moreover, the value of graphene carrier concentration is found to be correlated with the density of nanowires in contact with graphene. The lowest concentration of defects is observed for graphene deposited on nanowires with the lowest density. The contact between graphene and densely arranged nanowires leads to a large density of vacancies. On the other hand, grain boundaries are the main type of defects in graphene on rarely distributed nanowires. Our results also show modification of graphene carrier concentration and strain by different types of defects present in graphene. Therefore, the nanowire substrate is promising not only for strain and carrier concentration engineering but also for defect engineering.

## Introduction

The combination of excellent electrical and mechanical properties with interesting physical phenomena occurring in two-dimensional structures makes graphene an interesting experimental material to study [1–3]. Importantly, it is a promising material for new kinds of low-dimensional transistors, gas sensors, ultra-capacitors, electrodes for solar cells, and for van der Waals heterostructures. In order to construct these devices, an interaction between graphene and adjacent layers should be studied. It is well established already that graphene grown on silicon carbide is less strained on substrate terraces than on terrace edges, while electron concentration on the edges is lower than that on terraces [4]. This example shows that fluctuations of substrate morphology substantially modify graphene properties.

A novel approach of graphene-based nanostructures are van der Waals heterostructures in which graphene is transferred onto another material with a different morphology and electronic properties [5]. However, in those kinds of structures several aspects, such as strain induced by mechanical contact between materials or gating of graphene by neighbouring layers, are important for further applications. Furthermore, electron scattering on defects modifies graphene properties in several ways, for example, additional scattering centres reduce carrier mobility and, consequently, graphene conductivity. On the other hand, chemical functionalization of graphene may improve the sensitivity of graphene-based sensors [6]. Therefore, the control of density and types of defects in graphene might be a new way to prepare efficient molecular sensors.

Systems containing graphene on nanowires have been used in solar cells to increase their efficiency. In particular, it has been shown that the application of nanowires in solar cells decreases light reflection by scattering of light in between nanowires [7–8]. Nanowires have also a high cross-section of light absorption [9]. However, the interaction between corrugated nanowire substrate and graphene could substantially increase the scattering of carriers in a graphene electrode and decrease its conductivity. Therefore, detailed studies of the interaction between nanowire substrate and graphene are crucial to gain a deep understanding of the phenomena occurring on such interface.

One of the most common experimental techniques for studying properties of graphene is Raman spectroscopy [10]. Non-invasive measurements of inelastic light scattering give an insight into the phonon structure of graphene. The analysis of graphene G and 2D band parameters provides information about the number of graphene layers, strain, and carrier concentration [11–15]. Furthermore, in defected graphene, D and D’ defect bands are also observed and their intensity values are related to the concentration of defects and their types [16–20]. Thus, careful statistical studies of Raman spectra allow to determine how the substrate impacts graphene properties and, consequently, modifies the efficiency of graphene-based structures.

In this paper, we present detailed statistical studies of Raman spectra of graphene deposited on gallium nitride nanowires (GaN NWs) with different variations in height. The electric field induced in GaN predicted by theoretical calculations could reach 5 MV/cm [21]. This is an effect of high spontaneous and piezoelectric polarisations in the wurtzite structure of GaN. Consequently, a high concentration of carriers on the GaN surface can be observed [22–23]. Previous studies of graphene on GaN NWs have shown that electric charges located on the top of the GaN NWs strongly impact Raman scattering in graphene, causing an enhancement of the spectrum [24–25]. Therefore, studies of graphene on NWs with different densities and variations in height might give information about the role of supporting points on graphene properties. For example, the analysis of graphene deposited on uniformly distributed silicon nanopillars showed the dependence of graphene strain on the distance between the nanopillars [26]. For small distances, graphene was clearly suspended while graphene ripples caused by strain in the samples with larger distances between pillars were observed. Nevertheless, nanowire substrates could also gate graphene and affect carrier concentration and its distribution in the layer. Coulomb interaction between GaN NWs and graphene could also create vacancies in graphene and, consequently, increase the density of defects. In turn, as reported recently, strain and carrier concentration can be influenced and modified by graphene defects as well [27–30]. Therefore, the determination of how nanowire morphology, nanogating, and Coulomb interaction impact graphene properties is important not only for basic research but also for future applications of these structures. This requires the determination of the influence of graphene interaction with the NW substrate on the graphene properties, which is the main topic of this work. Detailed statistical analysis of various parameters of Raman bands is necessary for proper interpretation of the results. This approach enables one to obtain a better description of the graphene/substrate interaction than that from a separate analysis of graphene strain, carrier concentration, and defects. The presented analysis is also important in the tracing of the interdependencies of the parameters which characterize graphene properties.

## Experimental

Monolayer graphene was grown by chemical vapour deposition (CVD) on a copper foil with methane gas as the precursor [31]. Next, graphene was transferred onto GaN NWs substrates. Due to low adhesive forces between graphene and corrugated substrates, the most common method to transfer graphene with the use of poly(methyl methacrylate) (PMMA) polymer could not be applied for the transfer onto NW substrates [31]. Therefore, we used stable orthogonal frames from polydimethylsiloxane (PDMS) polymer to stabilize the graphene during the transfer process [32]. The GaN NW substrates were fabricated by plasma-assisted molecular beam epitaxy (PAMBE) in N-rich conditions on (111) silicon substrates [33]. The application of different growth temperatures and growth times allowed to obtain nanowires with different variations in height [34]. In our experiment, we used three samples differing in NW substrate height and density. The samples were named N (from NWs) with the addition of a number representing their variations in height. The detailed parameters of the samples are included in Table 1. In the first sample (thereafter named as N0) the NWs had a similar height of approx. 900 nm and their average density was approx. 140 µm^−2^. However, they formed clusters containing several merged NWs. In the second sample (N100) the height of the NWs varied by approx.100 nm, from 300–400 nm, and the density of NWs was approx. 400 NWs·µm^−2^. In the third sample (N500) the average density of NWs was similar to that in the first sample – approx. 120 NWs·µm^−2^. However, in this sample, two distinct groups of NWs were observed – approx. 80% of them were 1 μm in height while approx. 20% reached 1.5 μm.

**Table 1:** Parameters of three investigated GaN NWs substrates.

NWs	N0	N100	N500

height variation (nm)	0	100	500
height (nm)	900	300–400	1000–1500
diameter (nm)	40	40	40
density of individual NWs (μm^−2^)	140	400	120
distances between individual NWs (nm)	80	50	90
density of NWs clusters (μm^−2^)	20	50	15
distances between clusters (nm)	250	150	260

The samples were studied by scanning electron microscopy (SEM) using a SU8230 Hitachi microscope equipped with an in-lens secondary electron detector at 5 kV electron beam voltage. The Raman spectra were collected by using a T64000 Horiba Jobin-Yvon spectrometer with a Nd:YAG laser operating at 532 nm wavelength as the excitation source, and with an objective with a magnification of 100× that allowed to obtain a spatial resolution of approx. 300 nm. The laser power was reduced to 3 mW in order to reduce the heating effect. The micro-Raman maps were collected with 100 nm steps with a few square micrometres of mapping area for each sample. The spectra were calibrated by using a reference sample of high-quality silicon.

## Results and Discussion

**Figure 1:**
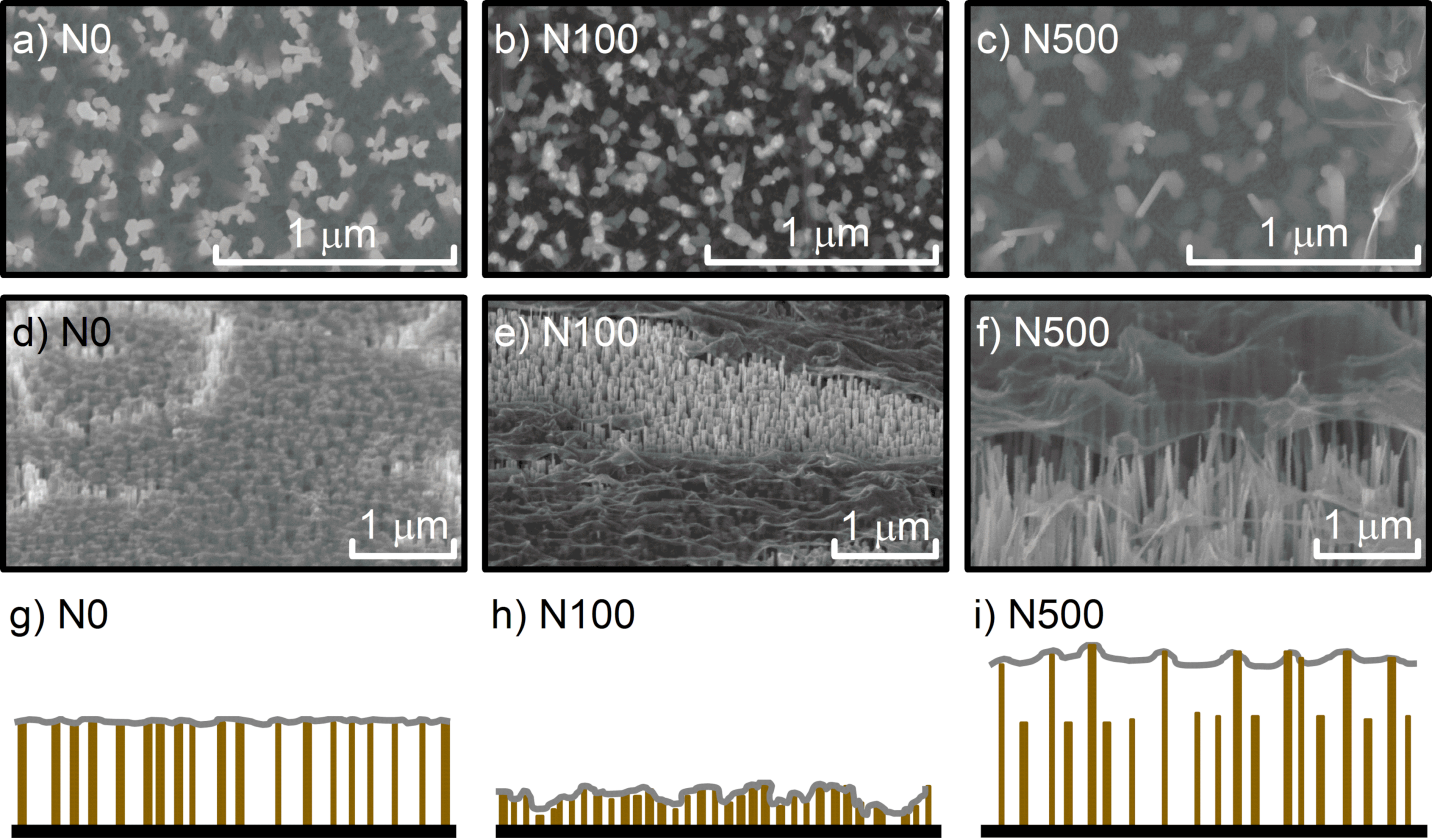
SEM images of graphene on GaN NWs with different variations in height in N0 (a,d), N100 (b,e), and N500 (c,f) samples. Images (a–c) were obtained at a 70° tilt of the sample while images (d–f) were collected in the plan view. The schematic profiles of the investigated samples are shown in (g–i).

The morphology of graphene deposited on NWs with different variations in height is presented in Figure 1.

The large cracks visible in the graphene layer are caused by the transfer process. Graphene on NWs with equal height is smooth (Figure 1a, Figure 1d). Small wrinkles are the evidence of a small expansion of the graphene hanging in between individual NWs. Larger wrinkles are observed in graphene on NWs with variations of 100 nm in height (Figure 1b, Figure 1e). Nevertheless, due to the higher density of supporting points, graphene is still attached to every single nanowire including those which are slightly lower in height. The most expanded graphene is observed in the N500 sample (Figure 1c, Figure 1f). Contrarily to the other samples, in this case graphene touches only the highest NWs and does not have any contact with the lowest ones. Furthermore, graphene in the N100 and N500 samples is pierced by some of the highest NWs. The topography of graphene on NWs with different variations in height is also visualized in the schematic profiles (Figure 1g–i). The SEM results suggest that both parameters, namely differences in height and density of NWs under the graphene, affect graphene morphology. Therefore, according to our previous results, a higher number of NWs in contact with graphene may increase the effect of nanogating while a lower number of supporting points could increase graphene strain [25–26].

**Figure 2:**
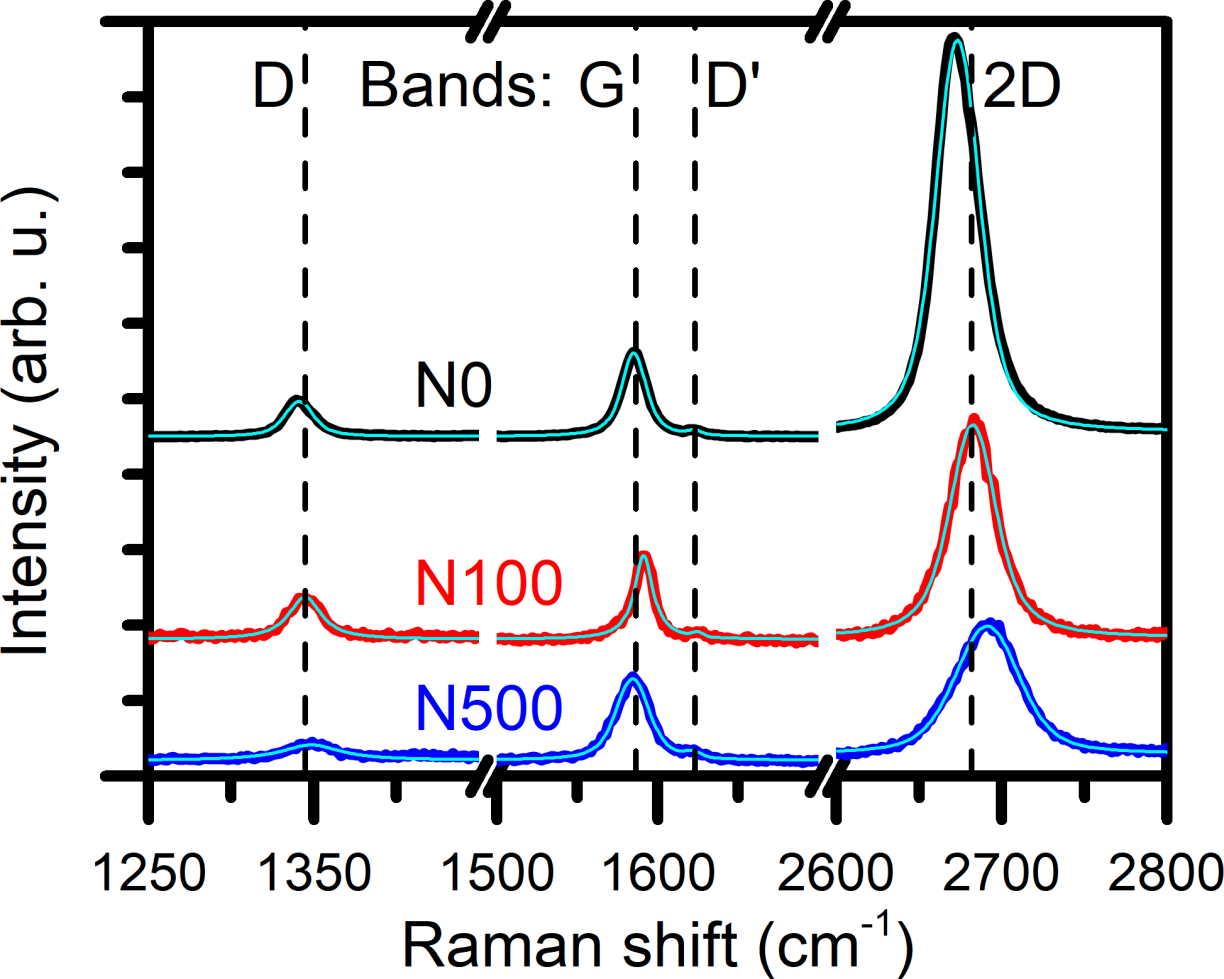
Representative Raman spectra of graphene on NWs with different variations in height normalized to the G band intensity. Light blue lines are fitted curves.

The analysis of representative Raman spectra for each sample shows that both graphene bands (G band at approx. 1585 cm^−1^ and 2D band at 2680 cm^−1^) and both defect bands (D band at 1345 cm^−1^ and D’ band at 1620 cm^−1^) are observed (Figure 2).

In order to recognize how NWs locally modify graphene strain, carrier concentration, and defects, a statistical analysis of band parameters over the whole Raman micro-mapping area was performed. In our system the lowest mapping step is comparable with the average distance between NWs and three times smaller than the diameter of the laser spot. Therefore, a single measurement is averaged over a few NWs and local interactions between graphene and small groups of NWs are traced rather than interactions with a single NW. The graphene strain can be studied by the analysis of the position of the 2D band energy and its full width at half maximum (FWHM). The dependence of graphene strain on the 2D band energy shift is described by Equation 1 [35]:

[1] $$E_{2\text{D}}=E_{2\text{D}}^{0}-2\gamma_{2\text{D}}E_{2\text{D}}^{0}\Delta \varepsilon ,$$

where γ_2D_ is the Grüneisen parameter, Δε is a value of strain, and the value of 2D band energy for unstrained graphene 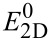 was reported to be 2677.6 cm^−1^ [14]. Positive values of Δε correspond to tensile strain while negative values correspond to compressive strain. The Grüneisen parameter determines the change rate of a given phonon frequency in a crystal with respect to strain. Its value depends on the strain type and substantial differences between values of the Grüneisen parameter for uniaxial and biaxial strain were observed [14,36–39]. Thus, a description of strain in the structure of graphene deposited on a large number of supporting points is not straightforward. Consequently, we cannot calculate the absolute value of strain; however, its qualitative description is still possible. The 2D band has a complex line shape due to the double resonance signal [40–41]. Therefore, the graphene strain could be qualitatively examined by the analysis of the 2D band FWHM [14,42].

**Figure 3:**
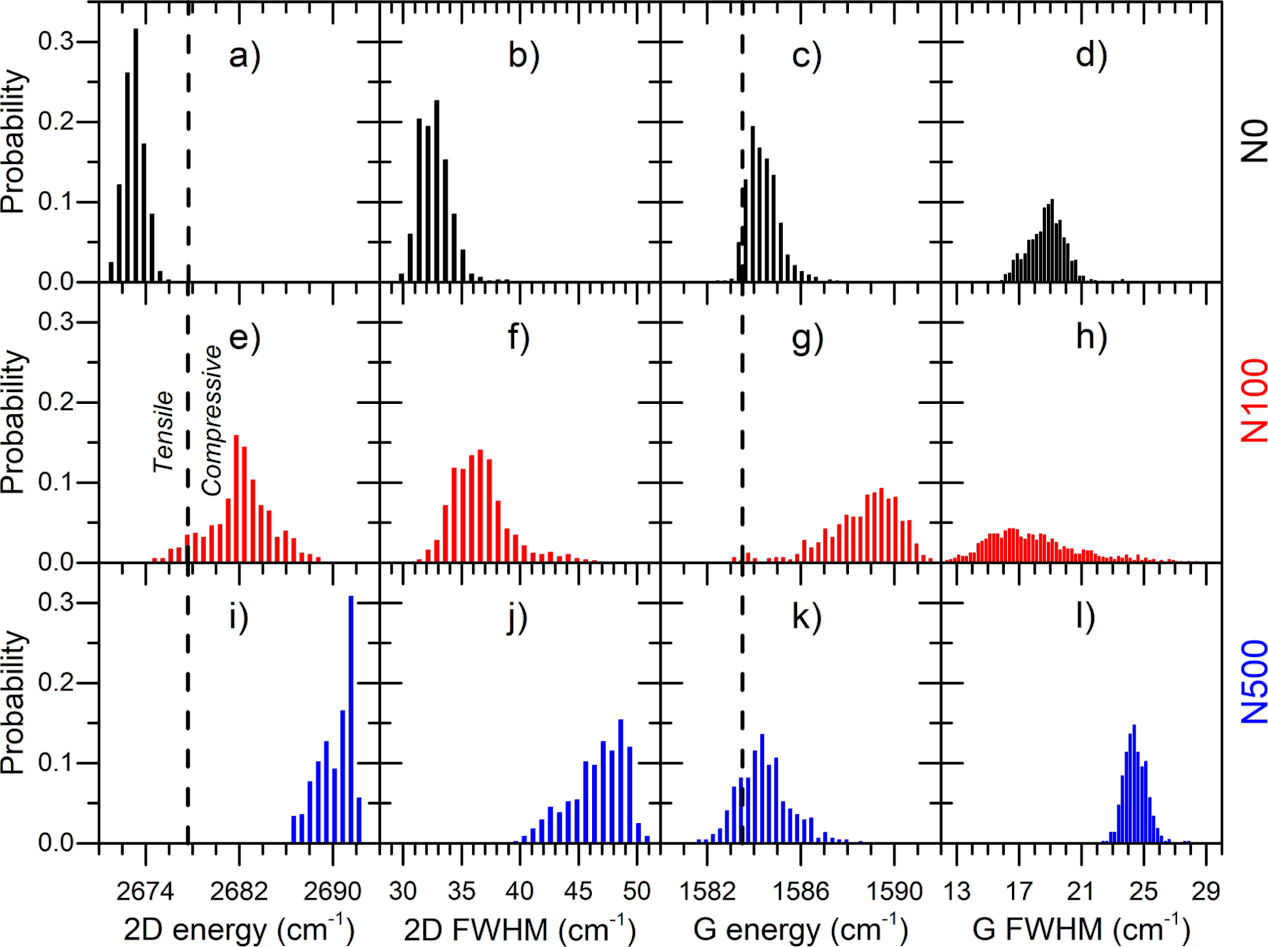
Histograms of 2D band energy (a,e,i), 2D FWHM (b,f,j), G band energy (c,g,k) and G FWHM (d,h,l) for N0, N100, and N500 samples, respectively. Dashed vertical lines correspond to 2D and G band energy values for unstrained and undoped graphene obtained from the literature [14].

The histograms of 2D band energy are presented in Figure 3a,e,i, while the calculated average values of 2D band energy and their standard deviations are presented in Table 2. Interestingly, for graphene transferred onto NWs with equal height (N0 sample), the strain has a tensile character (Figure 3a), while in graphene on NWs with different variations in height (N100 and N500 samples) the strain is rather compressive (Figure 3e,i). The highest value of average 2D band energy (2690.2 cm^−1^) is observed for the N500 sample, while the highest standard deviation of 2D band energy (2.7 cm^−1^) is observed for the N100 sample (Table 2). Therefore, we can conclude that the highest strain in the N500 sample is related to the extension of graphene between rarely arranged supporting points while the highest local strain fluctuations are observed for graphene transferred onto densely arranged NWs with medium differences in height (N100 sample). We suppose that the strain in the graphene on NWs is uniaxial or biaxial only in a local scale, between the nearest NWs.

**Table 2:** Average 2D and G band energy values (<*E*_2D_> and <*E*_G_>) with their standard deviation (σ*E*_2D_, σ*E*_G_), 2D and G band FWHM (<*F*_2D_> and <*F*_G_>) with their standard deviation (σ*F*_2D_, σ*F_G_*) and calculated value of the average strain (Δε) in graphene on NWs with different variations in height. Positive or negative values of graphene strain correspond to tensile and compressive strain, respectively.

	N0	N100	N500

<*E*_2D_> (cm^−1^)	2673.0	2682.1	2690.2
σ*E*_2D_ (cm^−1^)	0.9	2.7	1.5
Δε (%)	+0.07	–0.07	–0.20
<*F*_2D_> (cm^−1^)	32.3	36.6	46.6
σ*F*_2D_ (cm^−1^)	1.3	2.5	2.3
<*E*_G_> (cm^–1^)	1584.4	1588.6	1584.4
σ*E*_G_ (cm^–1^)	1.2	1.6	1.1
<*F*_G_> (cm^–1^)	18.8	17.9	24.5
σ*F*_G_ (cm^–1^)	0.7	3.0	0.7

However, in a Raman experiment, the excitation beam of 300 nm in diameter probes a larger area containing several NWs. Due to the random distribution of NWs, the total character of strain is neither simple biaxial nor uniaxial. Therefore, we cannot calculate the absolute value of graphene strain; however an estimation of its value and a comparison between samples is still possible. Table 2 presents the calculated average values of strain for all the samples using the Grüneisen parameter equals to 0.012 as obtained by Mohiuddin et al.[14]. Interestingly, the average absolute value of strain for N0 and N100 samples is the same and equals to 0.07%. It is three times lower than that for the N500 sample, in which the strain reaches 0.2%.

These results are further confirmed by the analysis of 2D band FWHM (Figure 3b,f,j). The average value of 2D FWHM for N0 and N100 samples is comparable, although slightly lower than that for the N0 sample. On the other hand, for the N500 sample, 2D FWHM is significantly higher. This result confirmed the presence of a higher strain in the N500 sample. According to the analysis of 2D energy, different values of 2D band FWHM for N0 and N100 samples cannot be explained only by the effect of graphene strain. It has to be caused by other reasons like, for example, carrier mobility. The 2D band energy and FWHM also depend on carrier concentration. However, their changes are significantly lower than those found for the G band FWHM, which will be discussed in the next paragraph [43].

The analysis of graphene G band parameters allows for one to trace how NW substrate impacts carrier concentration. The G band energy dependence on carrier concentration is described by the Equation 2:

[2] $$E_{\text{G}}=E_{\text{G}}^{0}-2\gamma_{\text{G}}E_{\text{G}}^{0}\Delta \varepsilon +n\cdot 7.38\cdot 10^{13},$$

where γ_G_ is the Grüneisen parameter for the G band and *n* is carrier concentration in cm^–2^ [35]. 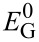 is the value of the G band energy for unstrained and undoped graphene which was found to be equal to 1583.5 cm^–1^ [14]. The sensitivity of the G band energy on the carrier concentration is caused by the presence of a Kohn anomaly near the Γ point in the phonon band structure of graphene [15]. Consequently, the G band energy significantly increases with an increasing concentration of both electrons or holes [44]. Unfortunately, the G band energy depends not only on the carrier concentration but also on the strain. Therefore, to estimate the value of carrier concentration in strained graphene, the analysis of the values of both G and 2D band parameters is necessary. Another parameter which depends on graphene carrier concentration is the FWHM of the G band [15]. The phonon lifetime is short in the case of a low value of the Fermi energy. Thus, the band width following the uncertainty principle consequently becomes larger. Increasing the Fermi energy values leads to an increase of the phonon lifetime and consequently to a decrease of the band width. In general, FWHM of the G band is positively correlated with the value of graphene strain. However, in the case of graphene with strain smaller than 0.2%, which is the case in our samples, such changes of FWHM are negligible [45].

The histograms of G band energy and its FWHM values are presented in Figure 3. The average value of the G band energy for N0 and N500 samples is the same and equals to 1584.4 cm^–1^ (Table 2), while for the N100 sample it is 4.2 cm^–1^ higher. A similar trend can be observed in the standard deviation of the G band energy. For the N100 sample it is significantly higher than that for N0 and N500 samples. On the other hand, the average G band FWHM value is similar for N0 and N100 samples and significantly lower than that observed for the N500 sample. Interestingly, the standard deviation of the G band FWHM for the N100 sample is more than four times higher than that for N0 and N500 samples. As it was discussed before, the existence of a medium tensile strain should decrease the value of the G band energy in the N0 sample. Similarly, the compressive strain observed in the N100 sample should increase the value of the G band energy. The analysis of the characteristic values of the Grüneisen parameters for different types of strain shows that the strain-induced change of the G band energy is less than two-times smaller than the change observed for the 2D band. However, values of the G band energy in N0 and N100 samples are approx. 3 cm^−1^ higher than what expected from the strain impact. Considering the low value of G band FWHM for both samples, changes of the G band energy in the N0 and N100 samples could be explained by the higher carrier concentration in these samples than that in the N500 sample. The lowest value of G band FWHM is present in the N100 sample, which suggests that this sample has the highest carrier concentration among all investigated samples.

Two factors should be taken into account when explaining our results. First, differences in height and density of the NWs impact graphene elongation and, consequently, affect graphene strain. Higher differences in NW height in the N500 sample increase graphene strain while a larger density of GaN/graphene supporting points in the N100 sample is responsible for the local reduction of strain. Second, GaN nanowire substrate modifies graphene carrier concentration by a self-induced nanogating [25]. The local carrier concentration in graphene on NWs is higher than that in graphene between NWs. A large number of NWs in contact with graphene in the N0 and N100 samples increases the value of carrier concentration. Our results also suggest that the low density of NWs contacting graphene in the N500 sample is responsible for the low value of carrier concentration. Therefore, the density of NWs supporting graphene could be responsible for the observed values of strain and carrier concentration. Moreover, high values of standard deviation for G and 2D band energy values and FWHM for the N100 sample is probably caused by local fluctuations of NW height in densely arranged NWs. Therefore, strain and carrier concentration in the N100 sample significantly change between data points. Moreover, a higher value of the 2D band FWHM in the N100 sample suggests different carrier mobility in N0 and N100 samples (Figure 3b, Figure 3f).

**Figure 4:**
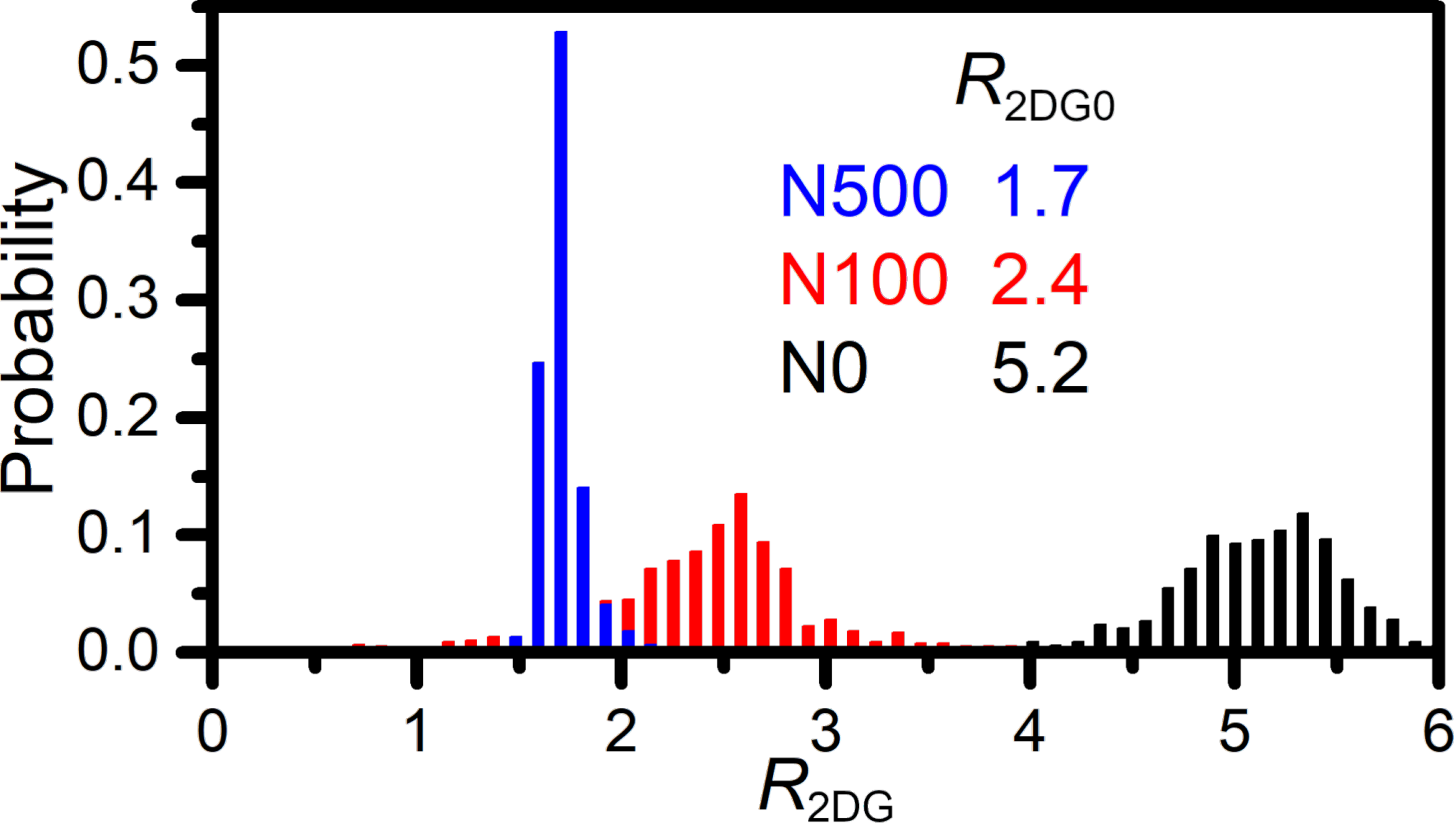
Histograms of intensity ratios between 2D and G bands.

The intensity ratio of 2D and G graphene bands in monolayer graphene has been reported to be negatively correlated with carrier concentration [15]. A higher Fermi energy increases the probability of scattering on free carriers, which adds to the scattering on phonons. Consequently, the intensity ratio of 2D and G Raman bands, *R*_2DG_, decreases when the carrier concentration increases. The histograms of *R*_2DG_ for all the measured samples are presented in Figure 4. The highest standard deviation is observed for the N100 sample, which is 1.5 times higher than that for the N0 sample and six times higher than that for the N500 sample (see Table 2). The average value of the *R*_2DG_ ratio is the highest for the N0 sample (5.2) and the lowest for the N500 sample (1.7). Surprisingly, the value of *R*_2DG_ suggests that the carrier concentration in the N500 sample is the highest of all the investigated samples, which disagrees with the conclusions obtained from the analysis of the G and 2D band energy values and FWHM.

**Figure 5:**
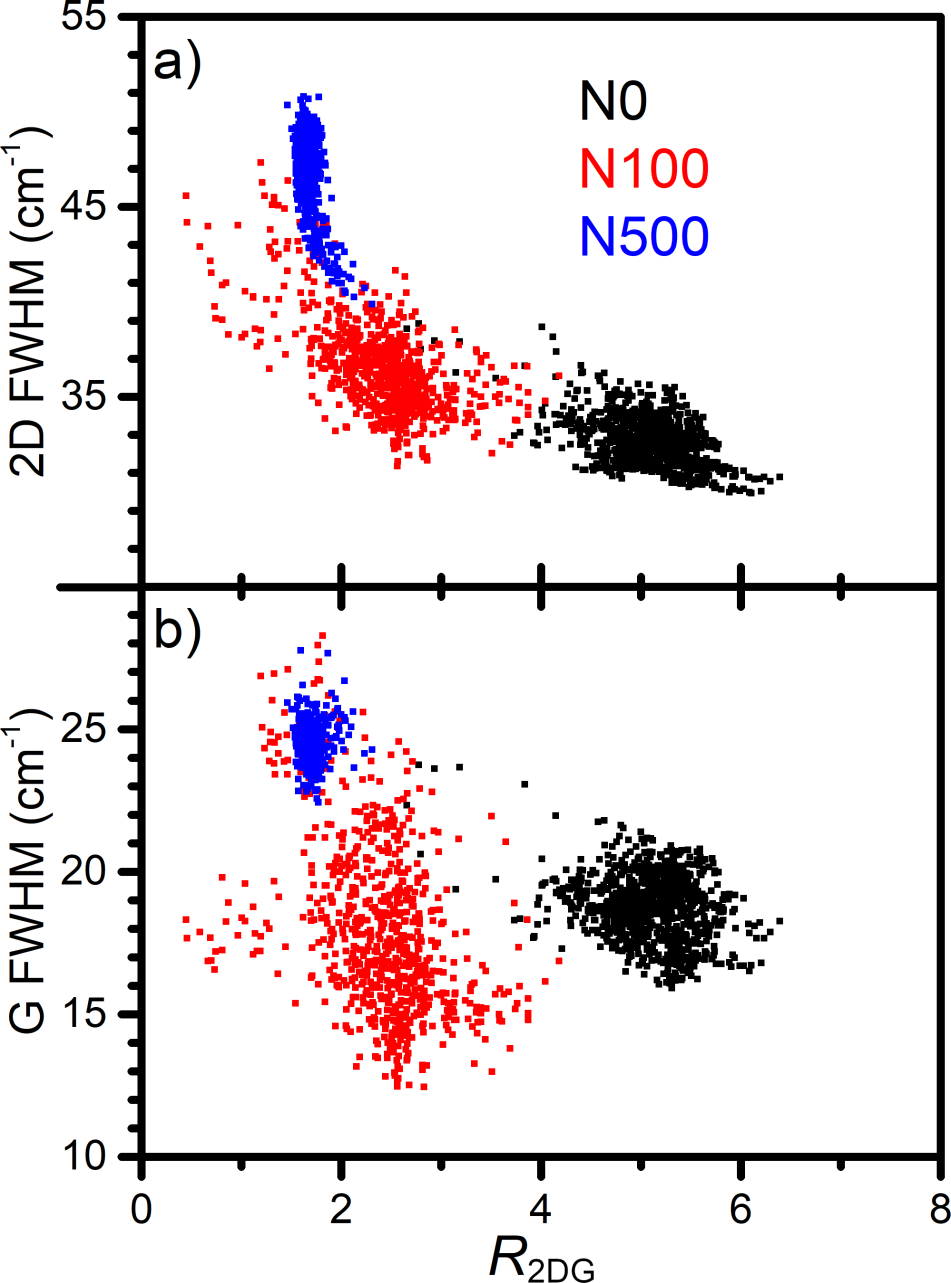
2D FWHM (a) and G FWHM (b) dependence on the ratio of 2D and G band intensities (*R*_2DG_) for graphene on NWs with different variations in height.

In order to clarify that contradiction, the analysis of the dependency of *R*_2DG_ on the strain and carrier concentration was performed by the analysis of 2D and G band FWHM. As discussed before, 2D band FWHM is positively correlated with the graphene strain, while G band FWHM is negatively correlated with the carrier concentration [14–15]. A negative correlation between *R*_2DG_ ratio and 2D band FWHM is observed (Figure 5a). Therefore, it can be concluded that *R*_2DG_ decreases when graphene strain increases. On the other hand, the analysis of the *R*_2DG_ dependence on G band FWHM does not show any evident correlation (Figure 5b). The experimental points for each sample are separated from each other. Thus, our results suggest that the intensity ratio of 2D and G bands in graphene on NWs is correlated rather with the strain than with the carrier concentration, which is in contradiction with the results reported by Das et al. [15].

Other kinds of graphene Raman bands visible in the spectra shown in Figure 2 are D and D’ bands – the so-called defect bands. In the case of graphene transferred onto NWs, the analysis of scattering on defects allows for one to trace how graphene structure changes after the deposition on NWs and how these changes depend on the density of NWs and their differences in height. An additional aspect is the impact of the defects on graphene strain and carrier concentration. Experimental studies have shown that some kinds of defects distort the graphene lattice and, consequently, increase graphene strain [46–47]. For example, the vacancies elongate graphene lattice and induce tensile strain, while Stone–Wales defects reduce bond length which results in compressive strain in graphene. On the other hand, a large number of vacancies may relax the strain in expanded graphene [27]. Additionally, disorder in graphene influences its carrier concentration. In the case of low density of defects, an increase of the disorder is correlated with an increase in the carrier concentration and the sign of charge carriers depends on the defect type [28–30]. For example, vacancies and nitrogen dopants in nitrilic and pyridinic positions introduce a p-type doping while nitrogen dopants in the graphitic position and hydrogen dopants in the pyridinic position result in n-type doping [48]. Thus, defect origin and density impact graphene strain and carrier concentration as well as the interaction with the substrate.

**Figure 6:**
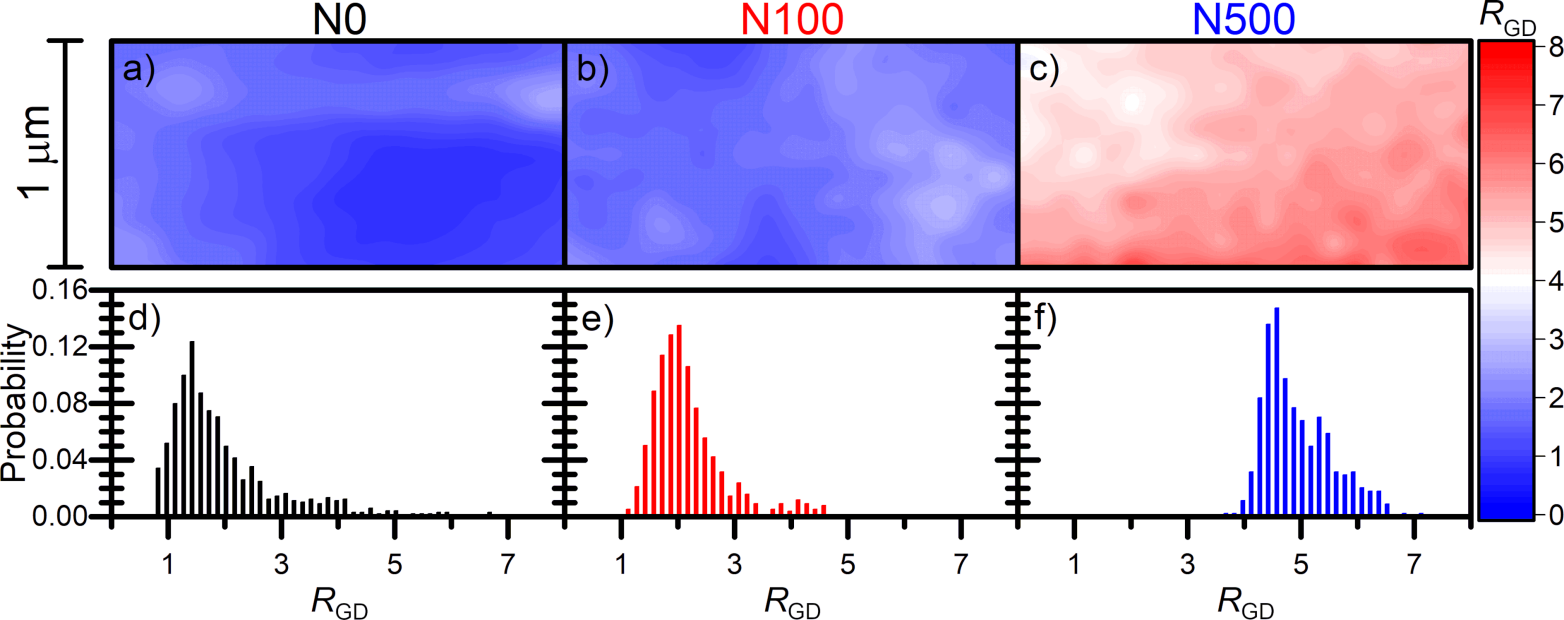
2D maps (a–c) and histograms (d–f) of the intensity ratio of the G and D bands (*R*_GD_) for all the three samples analysed.

The G band is generated by the scattering on iTO or iLO phonons near the Γ point of the Brillouin zone. For the presence of D band, the resonant scattering on the iTO phonon near the K point of the Brillouin zone and the defect are necessary. Consequently, the intensity of the G band is proportional to the laser spot area while the intensity of the D band depends on the number of defects in the excited area. Therefore, the density of defects *n*_D_ is inversely proportional to the intensity of the ratio of the G and D bands (*R*_GD_) and described by Equation 3 [19]:

[3] $$n_{\text{D}}(\mu {\text{m}}^{-2})=1.8\cdot 10^{14}{\left[\lambda_{l}(\text{nm})\right]}^{-4}R_{\text{GD}}^{-1},$$

where λ*_l_* is the excitation light wavelength. In order to visualize the distribution of defect density on the graphene surface, we performed spatial and statistical analysis of the intensity ratios of the G and D bands (Figure 6).

The respective 2D maps of the *R*_GD_ ratio are presented in Figure 6a–c. The distribution of the *R*_GD_ ratio in graphene on NWs with equal height is rather plain while in the N100 sample it is slightly modulated by the interaction with the NW substrate. A more evident modulation of the *R*_GD_ parameter is observed in the N500 sample. Figure 6d–f show histograms of the *R*_GD_ ratio while the average value of *R*_GD_ and the density of defects calculated using Equation 3 are presented in Table 3. The analysis of the histograms presented in Figure 6d and Figure 6e shows that the average value of *R*_GD_ and the width of the distribution are comparable in the N0 and N100 samples (Table 3). The average density of defect distribution in N0 and N100 samples is approx. 977 and 936 defects per square micrometre, respectively, while the density of nanowires under graphene in the N0 sample is three times lower than that in the N100 sample. The average value of *R*_GD_ in the N500 sample is two times higher (Table 3). Consequently, the average density of defects is two times lower than that in the previous two samples and it is equal to 449 per square micrometre. This observation suggests that a very low density of supporting points (24 NWs per µm^2^) is correlated with the low density of defects. However, a similar number of defects in the N0 and N100 samples remains unclear. Although the density of defects is similar for N0 and N100 samples, a different distribution of the *R*_GD_ ratio, which reflects defect density on the surface, is observed (Figure 6a–c). In case the of graphene transferred onto NWs with equal height, clusters of NWs locally interact with graphene stronger than with areas between them, whereas a large density of NWs with medium variations in height introduces a modulation of defect density in the N100 sample. Moreover, a low number of supporting points in the N500 sample is correlated with a lower average density of defects. However, the *R*_GD_ ratio in the N500 sample is densely modulated on the mapping area and does not reflect the supporting NW pattern. This result suggests that the deformation of graphene hanging between rarely distributed NWs also creates defects which explains the *R*_GD_ behaviour shown in Figure 6c. Therefore, our results suggest that not only the contact between NWs and graphene but also graphene deformation itself create defects in graphene and influence their spatial distribution. A very low density of supporting NWs also decreases the number of defects in graphene.

**Table 3:** Average ratio of G and D bands (<*R*_GD_>), defect density (*n*_D_) and percentage defect identification in graphene on NWs with different variations in height. The most common defect in each sample is indicated by an asterisk.

	N0	N100	N500

<*R*_GD_>	2.3	2.4	5
*n*_D_ (µm^−2^)	977	936	449
grain boundaries	10%	4%	*98%
mixture of vacancies	2%	6%	2%
single vacancies	*88%	*79%	—
hopping defects	—	8%	—
sp^3^ defects	—	3%	—

**Figure 7:**
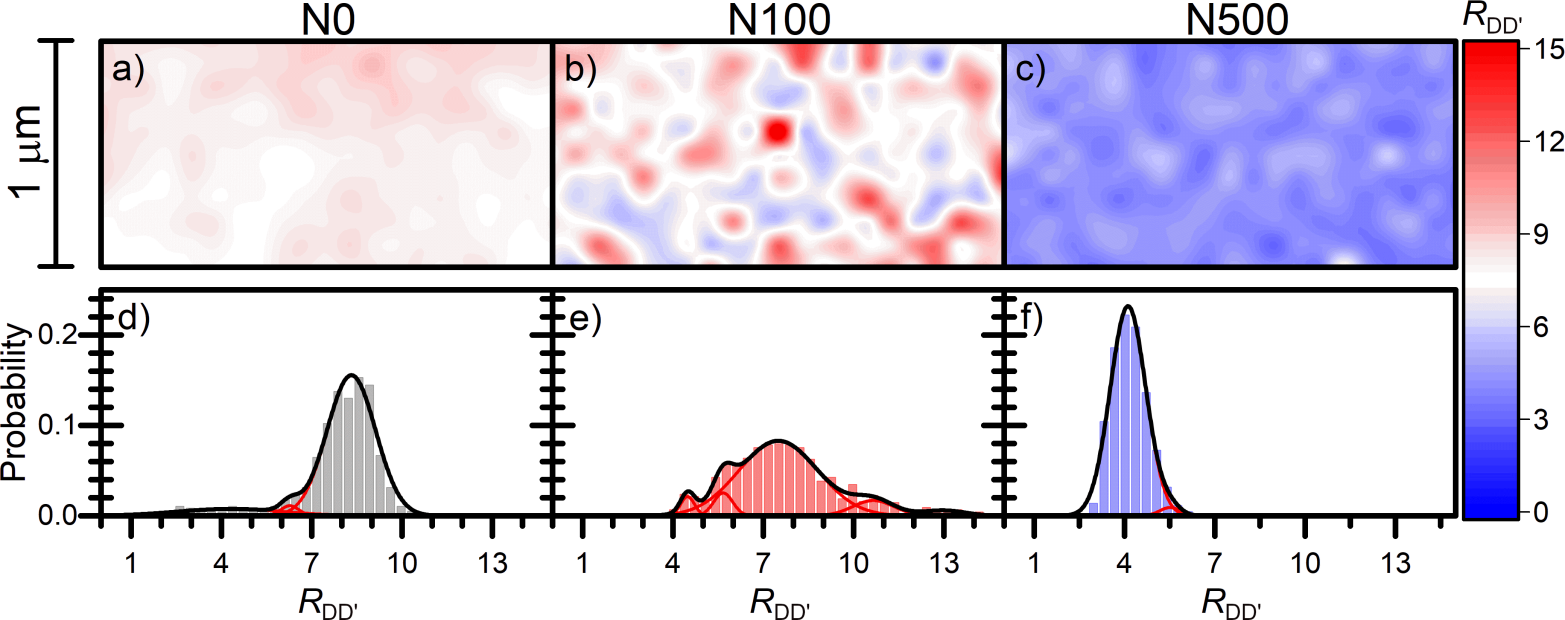
2D maps (a–c) and histograms (d–f) of the intensity ratio between D and D’ bands (*R_DD’_*) for all the three samples analysed.

The intensity of both defect bands D and D’ (*R*_DD’_) depends on defect density and parameters describing the perturbation introduced by the defects in the crystal lattice. These perturbation parameters depend on the type of defect and are different for the D and D’ bands. Thus, the intensity ratio between D and D’ bands characterize the type of defects in graphene [20]. Previous experimental results have shown that the value of *R*_DD’_ ratio equals to 3.5 is characteristic of grain boundaries, five is characteristic of multiple vacancies, seven corresponds to single vacancies, while 13 is observed for sp^3^ hybridisation defects [20,49]. Furthermore, theoretical calculations predicted values of 1.3 and 10.5 for on-site and hopping defects, respectively [50]. In order to identify the types of defects present in the studied samples, the intensity ratio between D and D’ bands was analysed (Figure 7). In contrast to the *R*_GD_ ratio, a strong modulation of *R*_DD’_ by the NW substrate is observed on the 2D maps (Figure 7a–c) for all the samples. This observation suggests that the nanowire substrate directly impacts the observed types of defects. The histograms of *R*_DD’_ ratio are presented in Figure 7d–f. The Gaussian distributions corresponding to the types of defects were fitted for each histogram. The percentage contribution of defects of a specific type for each sample were calculated by dividing the area of each Gaussian distribution by the sum of the areas of all fitted Gaussian distributions. The type of defects and their percentage contribution are included in Table 3. Interestingly, for all the samples, one maximum of high intensity and several smaller maxima can be observed, and approx. 80% or more defects are described by the main maximum. Single vacancies are dominant defects in N0 and N100 samples (maximum of distribution at *R*_DD’_ is equal to 8.3 and 7.5, respectively) while the grain boundaries are the main defects in the N500 sample (maximum of distribution at *R_DD’_* is equal to 4.1). At least 98% of all types of defects in the N500 sample are grain boundaries, which is a higher value than the obtained for vacancy contribution in N0 and N100 samples (88% and 79%, respectively). The standard deviation of the *R*_DD’_ ratio for the main maximum in the N500 sample is equal to 0.6, which is lower than that for N0 and N100 samples (0.8 and 1.3, respectively). Therefore, the interaction between graphene and rarely distributed NWs is more homogenous than that with densely arranged NWs. The largest number of different types of defects (i.e., five) are observed in the N100 sample, which confirms that graphene interacts with densely distributed NWs in a variety of ways.

**Figure 8:**
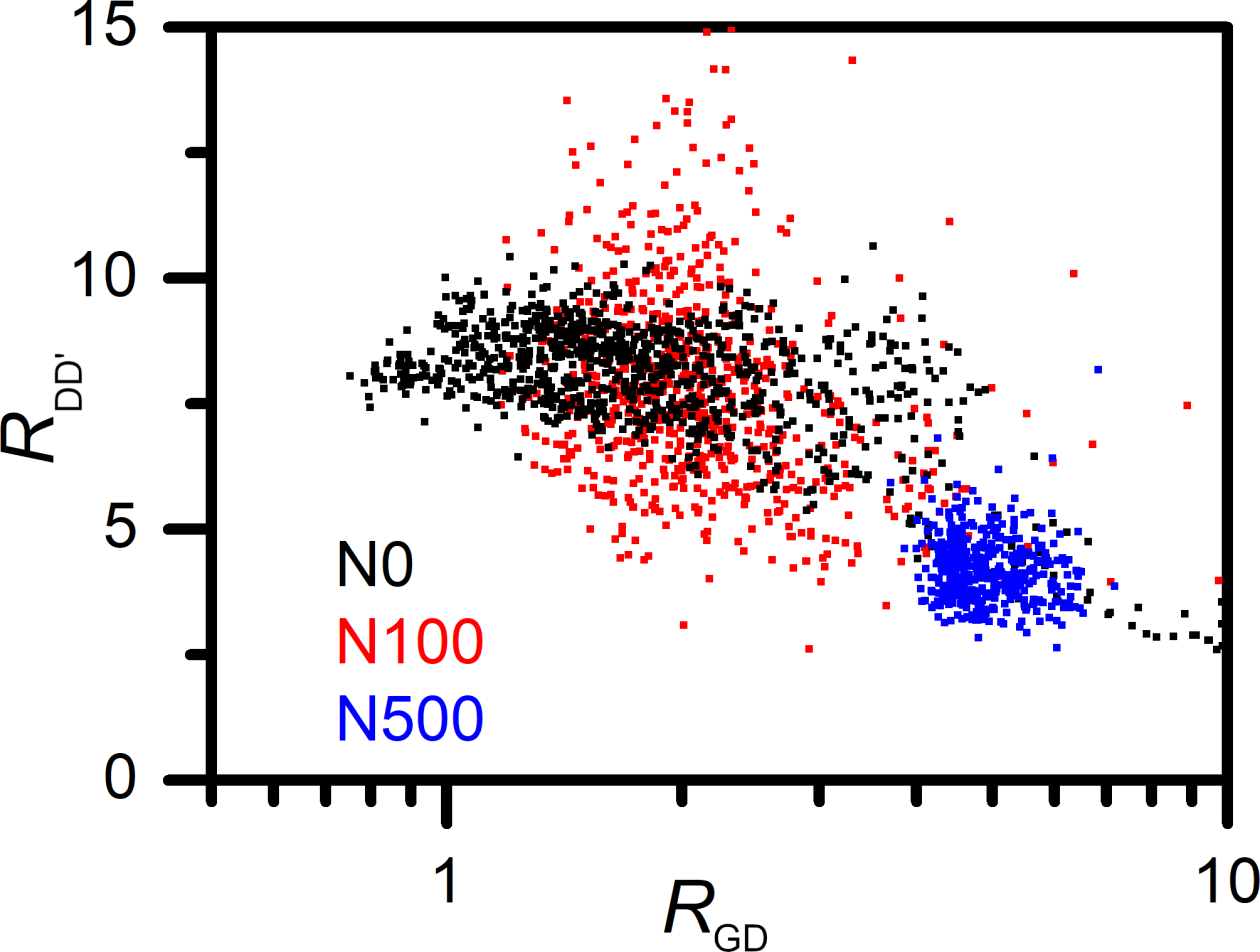
*R*_DD’_ ratio dependence on *R*_GD_ for graphene on NWs with different variations in height.

As discussed before, the analysis of the 2D band energy and *R*_GD_ ratio shows that N0 and N100 samples are characterized by a similar average absolute value of strain and a similar density of defects. In the case of the N500 sample, for which the strain is significantly higher, a higher value of *R*_GD_ and a lower value of *R*_DD’_ ratio were found. In Figure 8 we present the dependence of *R*_DD’_ on *R*_GD_ mapped points in the Raman experiment in all studied samples. A negative correlation of *R*_DD’_ and *R*_GD_ is observed. Therefore, the dependency of *R*_DD’_ and *R*_GD_ ratios on G and 2D band energy and FWHM were studied in detail in order to trace the interdependence between disorder parameters and carrier concentration or strain. No explicit correlations between *R*_GD_ and *R*_DD’_ ratios and carrier concentration were found. However, both parameters were correlated with 2D band FWHM and, consequently, with graphene strain for all the investigated samples. A local stretching of graphene observed in the investigated samples should rather elongate the graphene lattice than create new defects. Therefore, the lower density of NWs supporting graphene in the N500 sample is responsible for the lower density of defects and higher strain. The higher density of defects in the N0 and N100 samples is caused by the higher density of NWs under graphene. However, the reason for a different kind of strain (tensile/compressive) in these two samples is unclear. From the discussion above, the dependence between the density of NWs supporting graphene and the types of defects is nontrivial. Our results suggest that in graphene deposited on rarely arranged NWs, the grain boundaries are the most dominant type of defects. Densely arranged nanowire substrates introduce vacancies in the graphene deposited on them. Furthermore, the presence of a large number of vacancies in the N0 and N100 samples together with gating by the nanowire substrate could be responsible for increasing the carrier concentration, which is also confirmed by other studies [25,48]. In the N500 sample, in which most of defects are grain boundaries, less number of bonds were cracked. Consequently, the number of carriers is lower than in that in the samples with a significant presence of vacancies. Vacancies could also increase local tensile strain in graphene similarly as the nanowire substrate.

Therefore, high differences in NW height and low density of supporting points decrease the observed density of defects and highlight grain boundary defects omnipresent in the graphene layers. The contact with NWs of lower differences in height and higher density of supporting points creates more vacancies and increases their density on the surface. Moreover, graphene strain and carrier concentration can be locally modified by the different density of defects and their types. Thus, further studies on the influence of NWs supporting graphene and graphene strain, carrier concentration, and defects performed with higher resolution are essential.

## Conclusion

We transferred graphene onto GaN NWs with 0, 100, and 500 nm variations in height and studied their properties by SEM and Raman spectroscopy. Graphene on NWs with variations in height was rippled and pierced by the highest NWs. A detailed analysis of the Raman spectra showed that differences in NW height as well as NW density strongly impact graphene strain and carrier concentration. The highest strain value was observed for the sample with the highest differences in height (i.e., 500 nm). Unexpectedly, the strain in the graphene on NWs with equal height had a tensile character while the strain in the graphene on NWs with non-equal height had a compressive character. Analyses of G band energy and G band FWHM showed a positive correlation between the density of NWs under graphene and the value of its carrier concentration. In contradiction to previous reports, we found that the intensity ratio between 2D and G bands is correlated with the graphene strain rather than with its carrier concentration. Furthermore, analyses of *R*_GD_ and *R*_DD’_ ratios showed that the density of defects in graphene was affected by the nanowire substrate. Our results suggest that NWs supporting graphene with low differences in height introduce vacancies in the graphene. Increasing distances between NWs decreased the density of defects and exposed a larger number of grain boundaries omnipresent in any graphene layer. Furthermore, the vacancies could locally increase the graphene carrier concentration and tensile strain in the N0 and N100 samples together with the nanowire substrate. Thus, the density of NWs supporting graphene substrate and their differences in height impacted graphene carrier concentration and strain. It is, therefore, possible to consider the use of NW substrates for defect engineering in graphene and probably in other 2D materials.
